# Anxiety-related experience-dependent white matter structural differences in adolescence: A monozygotic twin difference approach

**DOI:** 10.1038/s41598-017-08107-6

**Published:** 2017-08-18

**Authors:** Nagesh Adluru, Zhan Luo, Carol A. Van Hulle, Andrew J. Schoen, Richard J. Davidson, Andrew L. Alexander, H. Hill Goldsmith

**Affiliations:** 1 Laboratory for Brain Imaging and Behavior, University of Wisconsin-Madison, 1500 Highland Avenue, Madison, WI 53705 USA; 2 Waisman Center, University of Wisconsin-Madison, 1500 Highland Avenue, Madison, WI 53705 USA; 3 Department of Computer Science, University of Wisconsin-Madison, 1210 West Dayton Street, Madison, WI 53706 USA; 4 Department of Psychology, University of Wisconsin-Madison, 1202 West Johnson Street, Madison, WI 53706 USA; 5 Department of Medical Physics, University of Wisconsin-Madison, 1111 Highland Avenue, Madison, WI 53705 USA

## Abstract

Anxiety is linked to deficits in structural and functional connectivity between limbic structures and pre-frontal cortices. We employed a monozygotic (MZ) twin difference design to examine the relationship between structural characteristics of the uncinate fasciculus (UF) measured by Diffusion Tensor Imaging (DTI) and anxiety symptoms in a sample of N = 100 monozygotic (genetically identical), adolescent twins. The MZ difference design allowed us focus on environmental factors that vary within twin pairs while controlling for genetic and environmental factors shared by twin pairs. Twins aged 13–18 years reported on symptoms of generalized anxiety and social phobia prior to participating in a neuroimaging visit. Regions of interest from the JHU ICBM atlas, including uncinate fasciculus and sagittal stratum as a control tract, were registered to the study template. We incorporated multiple diffusion tensor measures to characterize the white matter differences. Within twin pairs, the more anxious twin exhibited decreased fractional anisotropy (*t* = −2.22, *p* = 0.032) and axial diffusivity (*t* = −2.38, *p* = 0.022) in the left UF compared to the less anxious twin, controlling for age and gender. This study demonstrated the feasibility and advantages of adopting the MZ twin design for DTI measures in neuroimaging research.

## Introduction

Anxiety disorders represent one of the most prevalent adolescent mental health problems in the United States^1, 2^. Excessive anxiety can have a negative impact on relationships^3^, educational attainment^4^, and employment^5^. Anxious behaviors reflect individual differences in underlying neural function^6^. Non-invasive neuroimaging research in children and adults has attempted to uncover the neurological basis for anxiety disorders and symptoms^7^.

Diffusion tensor imaging (DTI) is an imaging modality that is highly sensitive to microstructural properties of biological tissues and the organization of the white matter (WM) structure^8^. It measures the three-dimensional diffusion of water according to the magnitude and orientation of the diffusion. Diffusion properties are modulated by the density and spacing of cellular membranes, cellular cytoskeleton, and the myelination of axons^8^. In white matter, the movement of water molecules perpendicular to the axon fibers is more hindered than in the parallel direction, resulting in more anisotropic diffusion. Thus, DTI measures have been used to characterize differences in WM microstructure for a broad spectrum of psychiatric conditions^9^. The most commonly investigated DTI measure is fractional anisotropy (FA), a derived scalar measure that is highly sensitive to the directional coherence of water diffusion. FA has been used as a quantitative indicator of WM integrity and connectivity, with lower values suggesting impaired WM integrity and decreased connectivity.

## Uncinate Fasciculus and Anxiety

Anxiety related behaviors are linked to increased bottom-up limbic activity^10, 11^ and decreased top-down prefrontal control^12, 13^. White matter tracts that connect the amygdala to the prefrontal cortex are a frequent target for neurological studies of anxiety^7^. The uncinate fasciculus (UF) is a hook-shaped association fiber that connects limbic regions (including the amygdala and anterior temporal lobe) with the orbital frontal cortex^14^. Structural features of the UF have been implicated in emotional regulation^15, 16^, attention bias^17^, and the integration of emotional states and cognition^18^.

Past attempts to link characteristics of the UF to psychopathology have produced mixed results. In keeping with a clinical framework that divides individuals into discrete diagnostic categories, previous research into WM integrity of the UF and its relation to anxiety has focused on clinical populations, typically Social Phobia (i.e. separation anxiety disorder), Generalized Anxiety Disorder (GAD), or Obsessive Compulsive Disorder (OCD)^19^. Compared to healthy controls, adults and adolescents with GAD had lower FA values in bilateral uncinate fasciculus (UF)^20–22^. Phan *et al*.^23^ reported lower FA values in the right UF among individuals with Social Phobia compared to healthy controls, while Baur^24^ reported lower FA values in the left UF among individuals with Social Phobia compared to healthy controls. Zarei *et al*.^25^ reported increased FA values in the right UF in adolescents with OCD compared to healthy controls. In contrast, Jayarajan^26^ reported no differences in FA values but increased radial diffusivity in the left and right UF in adolescents with OCD relative to healthy controls. A recent meta-analysis indicated that, in adults, FA values in the left UF is reduced across a variety of emotional disorders including depression, GAD, social phobia, OCD and post-traumatic stress disorder^27^.

The studies reviewed above tend to rely on discrete groups, usually with a focus on a specific diagnosis, with individuals having comorbidities being excluded. This approach has drawbacks. First, exclusions due to comorbidity limit the relevance of findings for clinical translation, as anxiety disorders are highly comorbid. A United States national survey on comorbidity reported that 60% of individuals with one anxiety disorder qualified for a diagnosis on a second anxiety disorder^28^. Further, anxiety disorders also share many behavioral features, such as intolerance to uncertainty^29^. Second, focusing on diagnostic categories excludes the less severe, subdromal levels of anxiety, which hold clinical relevance. Symptoms of anxiety are common, persistent, and varied in presentation. For example, prevalence rates for a Social Phobia *diagnosis* are around 9% for adolescents^2^, but many more non-diagnosed individuals report persistent *symptoms* of social phobia^4^. The prevalence rate for OCD is quite low at 2.3%, but as many as 30% of individuals report at least one symptom of OCD^30^. Many people with GAD refer to themselves as lifelong worriers^31^. Thus, anxiety is more trait-like than categorical.

A handful of studies have identified structural characteristics in the UF that are related to trait-like anxiety. Kim and Whalen^32^ first reported that FA values in a WM pathway between the amygdala and ventromedial prefrontal cortex, including part of UF, were negatively correlated with trait-like anxiety in healthy adults. A negative relationship between trait-like anxiety and FA values has been reported for children^33^ and adults^34, 35^. However, *positive* correlations have been also been reported between trait-like anxiety and WM integrity in the left UF in healthy adults^36, 37^. The extent to which variations in UF microstructure reflect experience-dependent influences has not been studied and may shed light on the variability in findings that have emerged. Thus, the MZ difference design is needed to resolve some of these conflicting findings.

## The MZ twin difference design

Neuroimaging studies typically use unrelated individuals and do not attempt to disentangle genetic and environmental influences on the neuroanatomical differences between subjects. Yet, several lines of research on individual differences in anxiety symptoms point to an etiology that includes both genetic and environmental factors^38^. In this context, “environmental” simply means “non-genetic”; environmental influences might be expressed via epigenetic processes. One widely cited meta-analysis estimates a heritability of 30–40% for general anxiety disorder^38^. Heritability is the proportion of phenotypic variation accounted for by genetic variation (where genetic variance is inferred from the resemblance of relatives rather than by directly measuring DNA variants). Other studies, including our own work, report slightly higher heritability estimates (50–55%) for common forms of anxiety^39–41^. The remaining, non-genetic variation is due to variation in environmental influences that affect one member of a twin pair but not the other^40^.

The MZ difference design is one way to probe experientially (i.e., environmentally) driven relationships between brain and behavior. MZ twins share 100% of their genes and, when reared together as in our study, 100% of their basic rearing or family environment. Thus, the MZ difference design rules out structural genetic factors (e.g. sequence variants, copy number variants) that contribute to individual differences as well as environmental factors that are shared by siblings reared together (despite sometimes varying *between* pairs). An example of these shared environmental factors would be the socio-economic status of a family that is shared by cotwins reared together.

Yet, twins, like all siblings, are also exposed to or seek out unique experiences or environments (i.e., experiential factors specific to one member of a twin pair) that contribute to their phenotype. The phrase “unique environment” does not mean environmental factors that are specific to an individual among the full sample; rather, “unique” means “not shared with the cotwin,” or unique to one twin within the pair. Examples of unique or non-shared environment relevant to anxiety might include experience of stressors affecting only one twin within the pair^42^, different treatment by family members or peers^43^, and different social roles within the family, as well as different illnesses and environmental exposures ^44^. Thus, MZ cotwin differences (or discordance in the case of dichotomous variables) are explained by unique environmental factors^45^.

A key advantage of the MZ difference design over the individual-level design is that the MZ difference design controls or quasi-controls for several factors that may moderate or otherwise obscure associations of anxiety and DTI measures in adolescents. For example, MZ twins are same-sex and same-age, and thus sex and age are already controlled for when examining MZ twin differences. Less obviously, the developmental timing of many shared events in the life histories of members of a pair is controlled. Especially relevant to MRI studies is the quasi-control over gross brain morphology that the MZ twin difference design affords. Variability in brain morphology is greatly reduced in MZ twins pairs (compared to matched non-twin controls) across a number of neurological measures^46^. Most importantly, this design controls for structural genetic variation between the co-twins. Thus, any differences found between the twins should be attributable to experience-dependent causes.

## MZ differences in uncinate fasciculus and anxiety

The strength of the MZ twin difference design as a tool to investigate neural correlates of psychiatric disorders was recognized decades ago^47^. Despite this, studies investigating WM integrity in common psychiatric disorders rarely employ an MZ difference design. den Braber *et al*.^48^ reported both increased FA and decreased FA values in a large number of white matter tracts in MZ twins who scored high on measures of OCD symptoms relative to their low-symptom cotwins. Hettema and colleagues^49^ conducted a pilot twin study (*N* = 34 female twins) comparing the DTI measures of adult twins who were diagnosed with generalized anxiety disorder with their non-affected cotwins. The twins diagnosed with generalized anxiety disorder had lower FA in the left UF and right inferior longitudinal fasciculus.

### Goals and hypotheses

**Figure 1:**
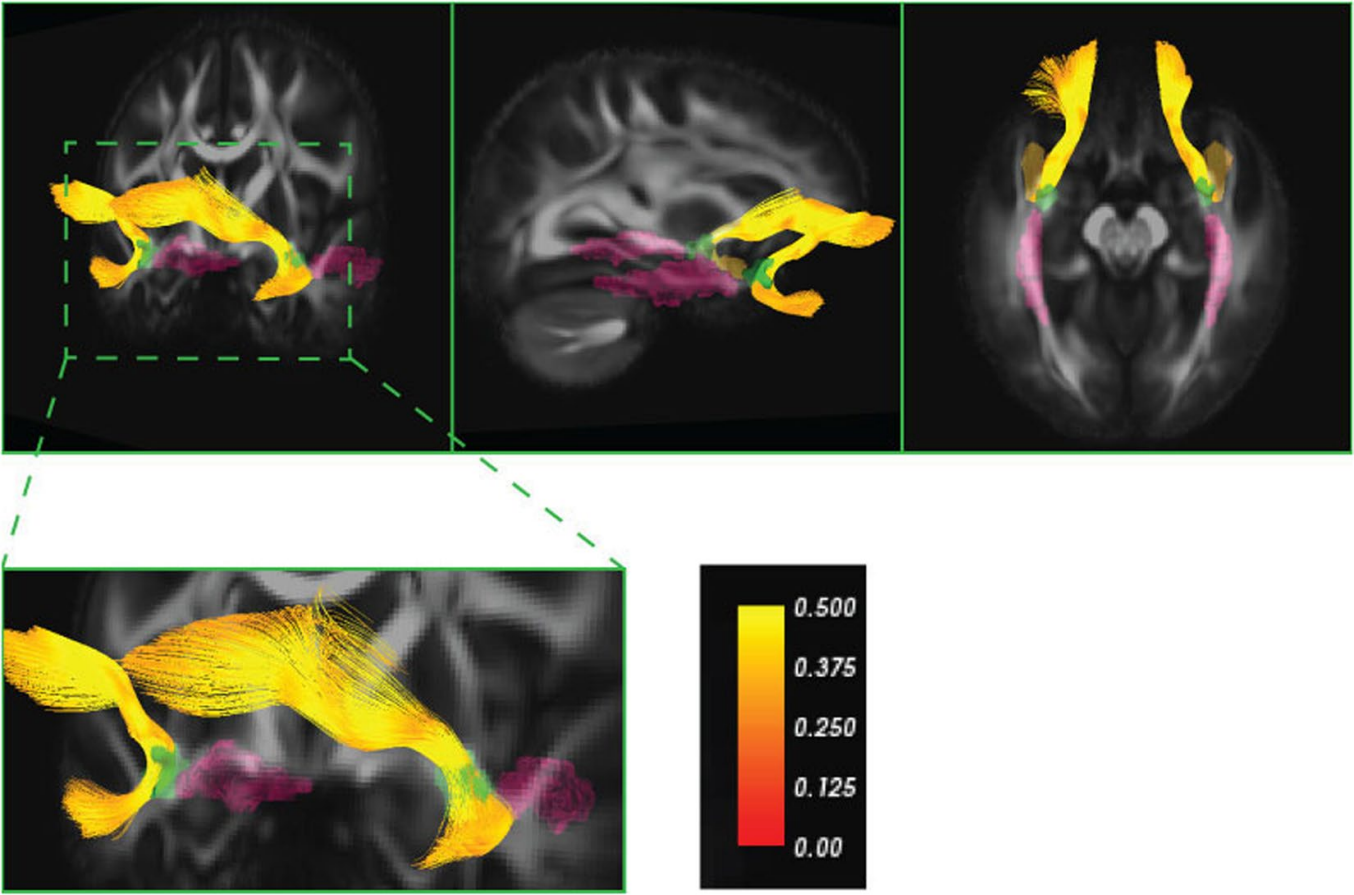
JHU ICBM atlas of uncinate fasciculus (UF) and sagittal stratum regions of interest are illustrated in green and pink, respectively. To better illustrate the relative anatomical location of JHU UF, deterministic tractography of UF is shown in yellow. The figure shows that UF tractography passes through JHU ROI, which corresponds to the intermediary segment of the UF between frontal and anterior temporal lobe.

We sought to address several gaps in the literature. First, studies of WM integrity and anxiety rarely include adolescent participants, despite anxiety related disorders being the most common class of disorders during adolescence. Second, most studies compare groups defined by diagnostic status, yet substantial evidence favors a trait-like approach rather than categorical taxa for psychopathology. That is, variation across individual traits or capabilities can be represented along a continuum representing greater or lesser degrees of health or adaptation, as in the NIMH Research Domain Criteria^50^. To address these two issues, we obtained self-reported *symptoms* of social phobia and generalized anxiety disorder from a sample of monozygotic (MZ) adolescent twins. Third, studies of unrelated individuals confound genetic and environmental influences. Employing an MZ twin difference design definitively rules out genetic effects and allows us to focus on environmentally influenced mechanisms. Fourth, the anatomical specificity of the UF may not be clear across studies due to the lack of clearly defined boundaries, because the UF is a long-range association fiber that can include temporal, intermediary, and/or frontal segments^19^. Here, we adopted a region of interest approach using the JHU ICBM atlas^51^, where the intermediary segment of UF is delineated between the temporal and frontal lobe (Figure 1). The JHU ICBM atlas of the UF and control sagittal stratum regions of interest are shown in green and pink, respectively. To better illustrate the relative anatomical location of JHU UF, we performed deterministic tractography of UF shown in yellow, using a previously published method^20^. Bilateral sagittal stratum in the JHU atlas (including inferior fronto-occipital fasciculus and inferior longitudinal fasciculus) was selected as a control tract due to its relative proximity to UF. Finally, to improve the specificity of the underlying WM differences, we incorporated multiple diffusion tensor measures, including AD, MD and RD.

The adolescent period is marked by developmental transformations across the prefrontal cortex and limbic brain regions^52^. Since UF is a WM pathway that continues to develop later in life, UF may be reflective of chronic experience-related changes in structure and function^53, 54^. Thus, we investigated how MZ differences for DTI measures of UF relate to MZ differences in anxiety symptoms. We hypothesized that, within twin pairs, the more anxious twin would show atypical WM microstructure measured by DTI in the UF, but not in a control tract. We had no hypothesis about bilaterality of effects.

## Results

Descriptive statistics are shown in Table 1. Our analyses focused on *symptoms* of anxiety; however, 36 participants (36%) qualified for a diagnosis of at least one anxiety or mood disorder. DISC symptoms counts were positively skewed. Adolescents reported an average of 3.1 out of 13 possible symptoms of Social Phobia and 2.9 out of 12 possible symptoms of General Anxiety. In contrast, very little skew was apparent in the HBQ social phobia and overanxiousness scales. On a scale where a 1 indicates almost no anxiety and 6 indicates a high level of anxiety, average scores fell in the middle −4.2 and 3.4 for social phobia and overanxousness, respectively. The right UF had significantly higher FA values than left UF in our sample (*t* = 3.1, *p* = 0.02).

**Table 1:** Demographic characterization of the sample, and mean levels of study variables.

Demographics	M (SD)			
Age	16.1 (1.7)			
% Female	54%			
% Right Handed	83%			
Mother’s Education (SD)	15.2 years (2.2)			
Father’s Education (SD)	14.8 years (2.2)			
Median Family Income	$80,000–$90,000			
**Anxiety measures**				
DISC generalized anxiety^a^	3.4 (2.6)			
DISC social phobia^a^	4.2 (4.1)			
HBQ overanxious^b^	2.9 (0.8)			
HBQ social anxiety^b^	3.1 (1.0)			
**DTI Measures**	**FA**	**MD (μm** ^2^ **/ms**)	**AD (μm** ^2^ **/ms)**	**RD (μm** ^2^ **/ms)**
L UF	0.456(0.032)	0.784(0.027)	1.221(0.046)	0.564(0.031)
R UF	0.471(0.034)	0.816(0.036)	1.289(0.052)	0.579(0.041)
L sagittal stratum	0.544(0.022)	0.859(0.035)	1.450(0.054)	0.557(0.034)
R sagittal stratum	0.539(0.021)	0.847(0.031)	1.420(0.479)	0.555(0.032)

^a^Symptom counts from the Diagnostic Interview Schedule for Children-IV.

^b^Likert rating (1–6) from the Health and Behavior Questionnaire.

### Twin Similarity

Table 2 shows the intraclass correlations (ICC) indexing cotwin similarity. The MZ twin intraclass correlation is an upper bound estimate of all familial influences (shared environment and genetic factors). The degree of twin similarity sets the context for examining intrapair twin differences. The upper bound heritability estimate for anxiety (0.67) is within the range of previously reported heritability estimates. Notably, up to 33% of the variance in anxiety can be attributed to non-shared experiential/environmental factors. In the lower section of Table 2, DTI measures show MZ twin intraclass Rs ranging from .51 to .75 for the various DTI measures in the left and right UF. These twin similarity correlations leave substantial variance to be explained by environmental factors not shared by pairs raised in the same home. Such non-shared variance is essential to the viability of the MZ difference design.

**Table 2:** Intraclass correlations* illustrating the degree of similarity of MZ twins on behavioral and DTI variables (N = 50 pairs).

Behavior Measures	ICC (R^2^)			
Anxiety Composite	0.67			
**DTI Measures**	**ICC (R** ^**2**^ **)**			
	**FA**	**MD**	**AD**	**RD**
L UF	0.66	0.51	0.56	0.60
R UF	0.75	0.57	0.57	0.67
L sagittal stratum	0.66	0.74	0.69	0.66
R sagittal stratum	0.71	0.77	0.69	0.80

All correlations significant at p < 0.05

### Individual-level analyses

We examined the ability of our anxiety measures to predict DTI measures from the UF considering each twin as an individual, while controlling for age, sex, and the dependence created by familial clustering. Using this individual-level design, we did not find any significant relationships between anxiety symptoms and DTI measures in the UF (top of Table 3) or control tract sagittal stratum (bottom of Table 3).

**Table 3:** Individual-level linear regression of neuroimaging measures on anxiety^a^.

	FA			AD			RD			MD		
	b	t	p	b	t	p	b	t	p	b	T	p
Left UF	−0.0043	−1.23	0.22	−0.0073	−0.13	0.20	0.0011	0.30	0.77	−0.0017	−0.50	0.63
Right UF	0.0021	0.59	0.56	0.0110	1.80	0.08	0.0002	0.04	0.97	0.0032	0.78	0.44
Left sagittal stratum	−0.0022	−0.85	0.40	0.0060	0.96	0.34	0.0048	1.23	0.22	0.0065	1.73	0.17
Right sagittal stratum	−0.0008	0.36	0.72	0.0005	0.09	0.93	−0.0006	−0.19	0.85	0.0005	0.17	0.87

^a^Linear regression includes covariates age and sex; standard errors account for clustering within twin pairs.

### Within MZ-pair analyses

**Figure 2:**
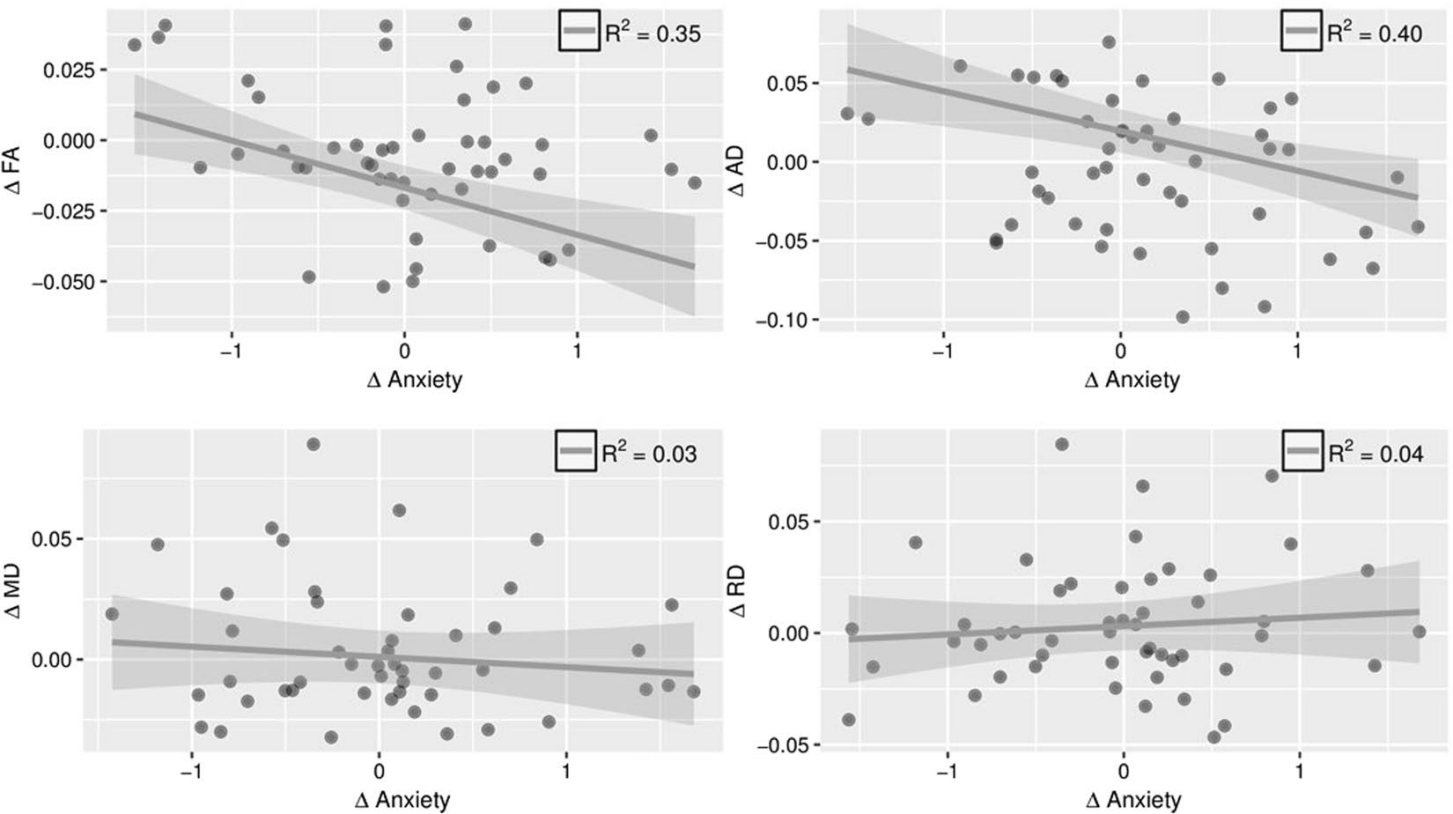
Intrapair anxiety differences are negatively associated with intrapair uncinate fasciculus differences in the left hemisphere FA (*t* = −2.22, *p* = 0.032) and AD (*t* = −2.38, *p* = 0.022). No significant relationships were observed for right UF.

Turning to the key analyses to test our hypothesis, we used the same framework as in the individual-level analyses but with intra-pair differences replacing the individual scores for both behavioral and DTI measures. These analyses control for genetic effects and quasi-control for other confounders, as explained in the Methods. These twin difference analyses show a significant negative relationship between anxiety differences and FA differences in the left UF, such that the more anxious twin had lower FA values than the less anxious co-twin (*t* = −2.22, *p* = 0.032, Figure 2). We also observe a negative association between anxiety and AD difference scores in the left UF (*t* = −2.38, *p* = 0.022, Figure 2). No relationship existed between anxiety and any DTI measures in the right UF (top of Table 4). Because FA is a normalized measure that takes into account all three eigenvalues of diffusion tensor, the observed relationship between anxiety and FA is likely driven by AD.

**Table 4:** Linear regression of twin differences in neuroimaging measures on twin differences in anxiety^a^

	FA			AD			RD			MD		
	b	t	p	b	t	P	b	t	p	b	t	p
Left UF	−0.011	−2.22	0.032*	−0.020	−2.38	0.022*	0.004	0.64	0.53	−0.005	−0.86	0.39
Right UF	0.002	0.46	0.65	0.008	0.81	0.423	0.001	0.13	0.89	0.003	0.48	0.63
Left sagittal stratum	−0.002	−0.09	0.53	0.004	0.44	0.665	0.007	1.27	0.21	0.024	1.59	0.12
Right sagittal stratum	0.002	0.52	0.60	−0.006	−0.81	0.422	−0.003	−0.69	0.49	−0.010	−0.77	0.45

Linear regression includes the covariates of age and sex of twin pairs.

**Figure 3:**
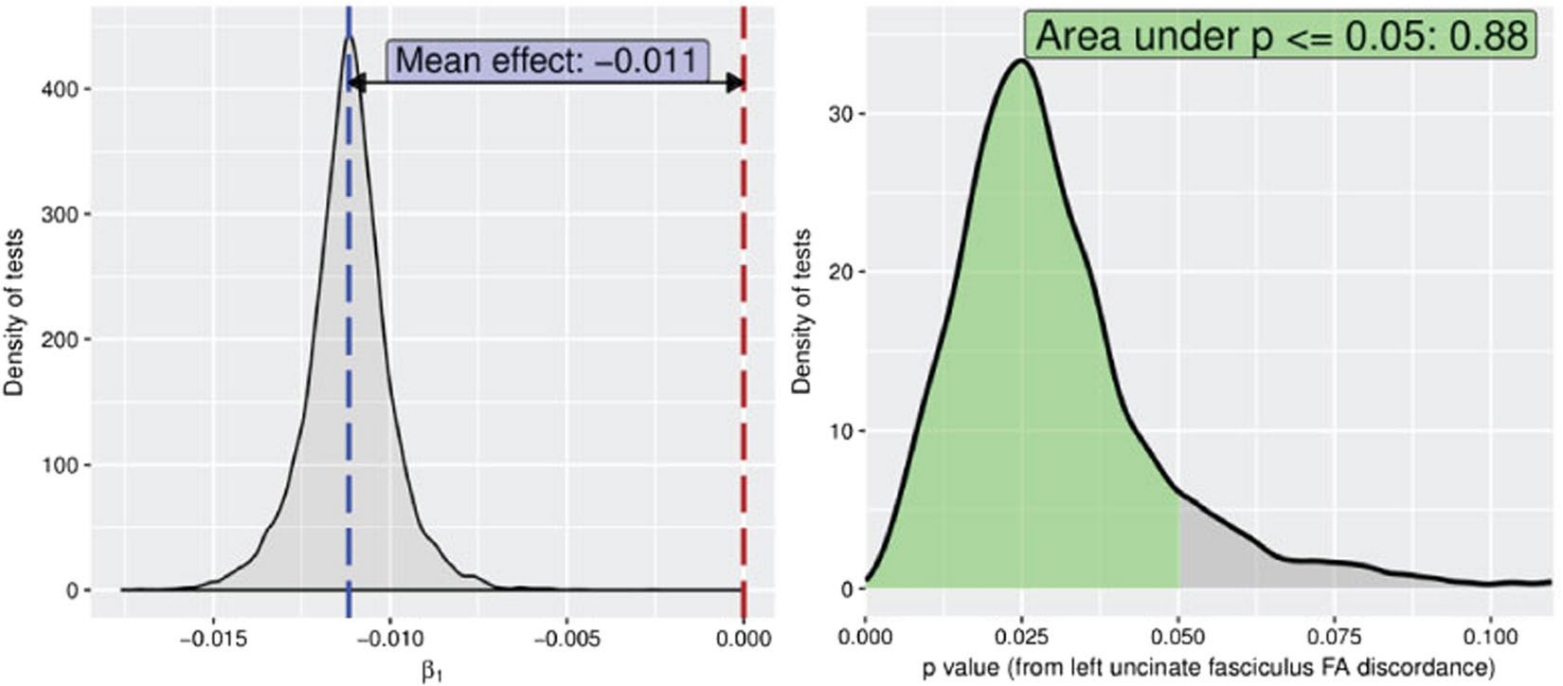
Distribution of beta (left panel) and *p* values (right panel) of association between anxiety and FA difference score after switching twin assignment 10^4^ times. 88% of the *p* values were lower than 0.05.

**Figure 4:**
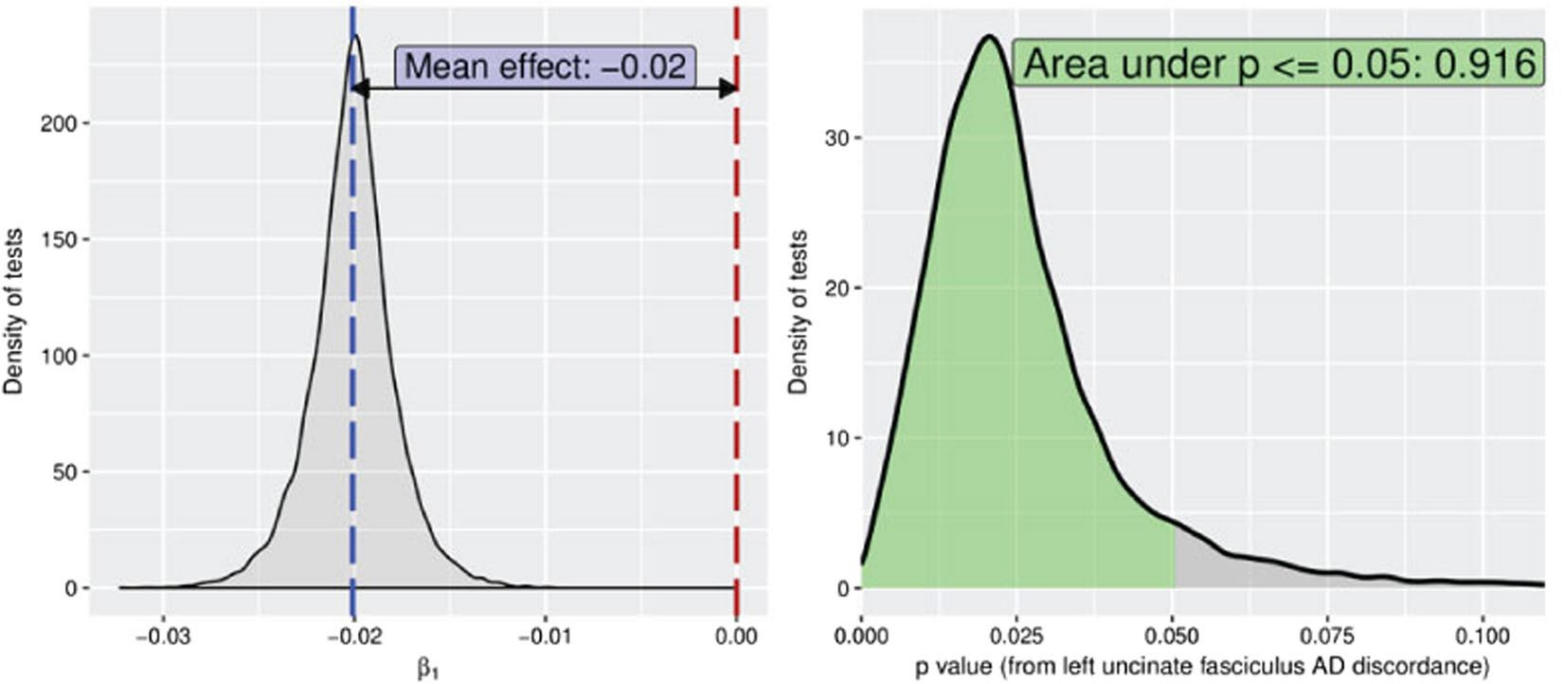
Distribution of beta (left panel) and *p* values (right panel) of association between anxiety and AD difference score after switching twin assignment 10^4^ times. 92% of the *p* values were lower than 0.05.

We conducted additional analyses to validate our findings and rule out a way that we might have capitalized on chance. First, we tested for an association between intrapair anxiety differences with intrapair differences in our control region, the sagittal stratum (bottom of Table 4). As predicted, no significant relationship was found. Second, to ensure that random assignment of twin order does not enhance the false positive rate of discovery, we generated the distributions of the *p* and β_1_ values by randomly swapping the Twin 1 and Twin 2 assignments 10^4^ times. These distributions are shown in Figs 3 and 4. Over 88% of the *p* values were lower than 0.05 for FA and 92% for AD, suggesting that the random twin assignment does not affect the statistical significance of findings when thresholded at *p* < 0.05. Third, the observed differences in anxiety and UF (FA and AD) are not associated with handedness.

## Discussion

The adolescent period is marked by rapid increases in cognitive capabilities but also a rise in mental health problems and difficulties related to emotional regulation^55^. These changes are due in part to developmental transformations across the prefrontal cortex and limbic brain regions^52^. A deeper understanding of anxiety disorders requires moving beyond behavioral correlates to elucidate the neural processes underlying the development of anxiety symptoms during this and other sensitive periods^50^.

We focused on white matter characteristics of the UF. The UF is plausibly related to anxiety symptoms given its structural and functional connections between areas of the limbic system and the pre-frontal cortex^16^. Anxiety symptoms may reflect a combination of exaggerated bottom-up reactivity and deficits in top-down control^13^. Altered white matter integrity in the UF could indicate impaired salience and emotional network connectivity in the limbic regions^27^ and may underlie abnormalities in emotion regulation.

One challenge of studying psychopathology is that a wide range of confounding factors, both genetic and environmental, can contribute to the observed variance, thus reducing the power to detect the relationship under investigation. Although some relevant confounders and demographic variables like gender and age can be controlled for, most research designs limit the number of confounders that can be considered. However, by studying MZ twins who are matched on both genetic makeup and shared environment, many unobserved confounding factors are naturally controlled for, and the remaining MZ twin difference can better illuminate the anxiety and WM relationship. Using simulated data, we further provided statistical arguments explaining why the MZ difference model can be more powerful (See Supplementary Materials). This likely explains why we only observed a significant relationship using the twin difference design but not the individual-level approach.

There are additional considerations on the type of traits that are most suitable for application of the MZ difference design. Low-to-moderately heritable traits, with moderate correlations between MZ cotwins, are most likely to yield positive findings. In other words, traits (e.g., anxiety) for which unique environmental effects are large are more likely to show correlations of those unique environmental effects with other measures. The low-to-moderate heritability of adolescent anxiety measures in the literature^38, 40^ thus suggests anxiety as a prudent choice for our analyses. In our sample, the ICCs (twin similarity correlations) for the left and right UF FA were 0.67 and 0.75, respectively, which is in agreement with ICC estimates in adult twin studies^56, 57^. Previous studies have reported that heritability estimates of UF FA increase with age from relatively low in early childhood to quite high in adulthood^56, 58^. In the current study, both samples were selected to ensure at least a portion of the twins were discordant for anxiety. Thus, the twin differences in behavior and brain structure may have emerged substantially earlier in development. With cross-sectional data, we cannot determine the timing of either twin differences in UF characteristics or twin differences in anxiety.

Because the MZ difference design quasi-controls for genetic influences on anxiety and white matter development, it is a powerful means of detecting experience-based influences on outcomes. We found that twin *differences* in FA and AD values in the left UF were negatively correlated with twin *differences* in anxiety. Notably, the more anxious twin was designated relative to the cotwin rather than the sample average. Thus, even subtle twin differences in the UF microstructure may predict differences in anxiety levels. By incorporating multiple DTI measures, we also found that the twin with higher anxiety showed a lower level of axial diffusivity (AD) in left UF, but not radial or mean diffusivity. AD, which correspond with the largest eigenvalue of the diffusion tensor, has been shown to be more sensitive to axonal structure than FA, the summary measure of microstructural integrity^59^.

Our results were specific to the left UF. Unfortunately, lateralization of brain structure in anxiety-related processes is ill-understood. However, our finding is consistent with two recent studies that observed an association of anxiety with decreased FA in left UF only^49, 60^. Zuurbier *et al*.^15^ also reported a positive correlation between emotional reappraisal and FA measured in the left UF. Moreover, Jenkins *et al*.^27^ performed a meta-analysis across studies of a wide range of emotional disorders and found that regions of reduced FA are most robust and replicable in the left hemisphere WM regions, including superior longitudinal fasciculus, UF, and anterior thalamic radiations (of note, significant clusters are present in both left and right hemisphere). Similar lateral differences are observed in functional MRI studies. For example, the left amygdala may be more active during emotional processing than the right amygdala in anxious individuals^61^.

Most studies on this topic employ a case-control design where cases have a full-blown disorder and controls are carefully screened for an absence of *any* history of psychiatric disorders. One-third of our participants qualified for a diagnosis of at least one anxiety or mood disorder, but, on average, participants endorsed few clinical symptoms of either generalized anxiety or social phobia on the diagnostic interview. In contrast, many participants reported moderate levels of subclinical anxiety on the HBQ. In other words, participants were more likely to experience anxiety that arises from typical day-to-day experiences than to experience clinically diagnosable levels of anxiety. This difference in the nature and extent of anxiety in our sample versus most other studies is important to consider in reconciling findings. At the least, our positive findings indicate that the association of UF integrity with anxiety extends outside the clinical range of anxiety.

### Limitations

We examined the anxiety and WM relationship using JHU atlas-based ROI in the uncinate fasciculus. Future studies can adopt full brain voxel-based analysis to maximize the spatial and anatomical specificity of anxiety and WM microstructure. Although full DTI metrics that are more sensitive to certain WM structures, these measures remain non-specific because a wide range of structural properties can modulate DTI values^8^. Briefly, MD and RD reflect the overall and lateral”size” of the diffusion tensors respectively, whereas FA and AD reflect how”elongated” the tensors are. Statistical differences in the elongation might exist despite similar sizes (one can think of having differences in the higher order moments instead of the means). The full mathematical treatment is provided in the supplement. Although deriving biological specificity from these DTI measures is tenuous, anxiety might not affect the overall “size” of the uncinate but nevertheless exert some effect on its elongation. Recent neuroimaging techniques, such as neurite orientation dispersion and density imaging (NODDI)^62^, may provide better specificity of underlying structure than the traditional tensor measures that we used.

Second, further research is needed to demonstrate the generalizability of our findings in non-twin populations. Twin pairs are more likely to experience birth complications and/or have low birth weight compared to singletons^63^. Similarly, Hulshoff Pol *et al*.^64^ reported significantly smaller total gray matter volume in second born twins and smaller total white matter volume in all twins compared to singletons. Even so, by late childhood twins are indistinguishable from singletons in terms of developmental outcomes^65^. As we noted earlier, most studies compare extremely discordant individuals (currently diagnosed vs. never diagnosed), whereas we focused on more normative levels of anxiety symptoms. Thus, our findings do not necessarily apply to overt anxiety disorders. Finally, most of our sample is white and middle class, which limits the generalizability to more diverse populations.

Third, our methods are correlational, with quasi-control of various confounding factors. Without longitudinal data or an experimental manipulation, we cannot determine if environmental influences on white matter development lead to anxious behavior, or, alternatively, if environmental influences on anxious behavior inhibit white matter development. However, we have confirmed that deficits in a structural component of the limbic system is associated with anxiety, and this association is dependent on unique environmental factors that are not shared by family members.

### Conclusions

This study aimed to demonstrate the feasibility and advantages of adopting the MZ twin design for DTI measures in neuroimaging research. Our results add to a growing literature that structural differences in MZ twins in the UF are uniquely related to measures of general anxiety and social phobia.

## Methods

### Participants

Participants were a selected subsample of 100 same-sex MZ twins from two community-based longitudinal twin studies^66^. Twins were selected for follow-up imaging if one or both twins were considered at-risk for developing anxiety, using the operational definitions of “at-risk” stated below.

#### Sample 1

Families of twins from the greater Madison, Wisconsin area were identified through state birth records and community outreach and invited to participate in an infant/toddler temperament study. Twins were assessed longitudinally at ages 6, 12, and 36 months, with some intermediate assessments on subsamples. Monozygotic twins (*n = *68) were selected for an adolescent imaging follow-up if one or both twins were identified as highly fearful of strangers^67^ or scored in the top 20% on an observational measure of object fear. The adolescent imaging sample was 93% white, 3% Hispanic, and 4% other. Mothers and fathers most frequently reported a college degree as their highest level of education. Median income was $80,000-$90,000 and mean age at imaging was 16 years (*SD* = 1.72, 13–19). Participants completed a questionnaire and a diagnostic interview 2 to 24 months prior to the imaging visit (average 7 months).

#### Sample 2

Families of twins from throughout the state of Wisconsin were identified though birth records and invited to participate in a research panel. At age 7 years, the full sample was mildly enriched for psychopathology (*i.e*., at least one member of the twin pair scored more than 1½ standard deviations above the mean level of parent-rated depression, anxiety, overanxiousness, oppositional defiance, aggression, conduct disorder, inattention, or impulsivity on the Health and Behavior Questionnaire – parent version, see below), and others were selected for low symptoms. Forty percent of enrollees met criteria for mild enrichment as described above, 25% scored below the mean on all behavior problem dimensions, and 35% were unselected cotwins. Participants were assessed at ages of approximately 7, 12, and 14 years.

Monozygotic twins (*n* = 56) were selected for an imaging follow-up if one or both twins reported chronic anxiety (i.e. scored above diagnostic threshold on one or more of eight anxiety/mood disorder sections from the Diagnostic Interview Schedule for Children-IV or the DISC Predictive Scales on two or more occasions). The imaging sample was 73% white, 11.5% Hispanic, and 11.4% other races/ethnicites. Mothers most frequently reported “some college” as their highest level of education (23%) and fathers most frequently reported a high school degree as their highest level of education (42.3%). Median family income was in the $80,000-$90,000 range. Average age at imaging was 16 years (SD = 1.7; range, 13–18). Twins completed a questionnaire packet and diagnostic interview 3 to 24 months before the imaging visit (average 12 months).

Questionnaires packets from both samples included a handedness questionnaire by Chapman and Chapman^68^, which asked which hand participants generally used for 13 routine activities, such as using a toothbrush or bottle opener. Participant responded with left hand, either, or right hand, which received scores of 1, 2, or 3, respectively. The 13 items were averaged, and participants were designated as right-handed if the mean score was above 2.

#### Combined Imaging follow-up

Exclusion criteria for imaging follow-up recruitment included the following conditions: cerebral palsy, claustrophobia, seizure disorder, metal orthodontic braces, dermal piercings, traumatic brain injury and developmental disabilities. A total of 108 twins with complete imaging data were included in the initial processing. Data from four additional twins were excluded from further analyses due to imaging artifacts, distortions, or excessive head movement, which also resulted in the exclusion of their co-twins. The final sample was *n* = 100 twins, 54% female, 75% non-Hispanic White, and 83% right-hand dominant. The University of Wisconsin–Madison Institutional Review Boards (IRB) approved all study protocols. Informed consent and assent were obtained from both twins and their legal guardian, and research methods were carried out in accordance with the approved guidelines.

### Anxiety Behavioral Symptom Measures

We created a composite measure from symptoms of social phobia and generalized anxiety disorder. Social phobia is characterized by avoidance of or intense discomfort in social situations. Generalized anxiety is characterized by excessive worry that is not focused on a specific situation or object and not tied to any specific stressful event.

Trained interviewers administered the Diagnostic Interview Schedule for Children-IV (DISC-IV)^69^ to adolescent participants during a phone interview. Adolescents also reported anxiety-relevant behaviors using the MacArthur Health and Behavior Questionnaire (HBQ)^70^. Respondents were given a choice of two opposing options that were stated in equivalent ways (e.g., *I worry about things I’ve done* vs. *I don’t worry about things I’ve done*). Respondents first selected the option that was most true for them and then rated it on a scale from 1 (*sort of like me*) to 3 (*really like me*). Each item was then converted to a 1 − 6 Likert scale with higher scores indicating more severe anxiety.

Standardized symptom counts from the Social Phobia and General Anxiety sections of the DISC-IV were standardized and averaged with standardized scores on the HBQ overanxiousness and social anxiety subscales. We omitted OCD symptoms from these analyses because the OCD symptom counts on the DISC-IV were highly skewed (average < 1 symptom), and the HBQ does not query obsessive or compulsive behaviors.

### Imaging Acquisition and Processing

Twins from both samples underwent identical MRI protocols on a 3.0 Tesla GE SIGNA (Discovery MR750) scanner with an 8-channel array head coil. Diffusion tensor imaging (DTI) was performed using a diffusion-weighted (DW), spin-echo, echo-planar imaging (EPI) sequence with 48 non-collinear encoding directions at DW b = 1000s . mm^−2^. Eight additional non-DW (b = 0 s . mm^−2^) images were acquired as reference volume. Other protocol parameters were TR/TE = 8000/66.2 ms; parallel imaging (ASSET with acceleration = 2); flip angle = 90°; isotropic 2 mm resolution (128 × 128 matrix with 256 mm field-of-view). Seventy-four contiguous slices (2 mm thick) were prescribed axially, covering the entire brain. Structural T_1_-weighted and functional BOLD imaging scans were also acquired but were not used in this study.

All processing was conducted on the combined sample. Tools from the FSL software package were used to correct for eddy current related distortions and head motion^71^. Image distortions from magnetic field inhomogeneity were corrected using field maps^72^. Estimation of the diffusion tensors at each voxel was performed using non-linear tensor estimation in the CAMINO software package. Diffusion tensor image registration tool DTI-TK was used for spatial normalization. This software performs white matter alignment using a non-parametric, highly deformable, diffeomorphic (topology preserving) registration method that incrementally estimates its displacement field using a tensor-based registration formulation. DTI-TK is the best performing in the class of diffeomorphic tensor registration algorithms^73^. Spatial normalization is first performed within each pair before mapping them to the population average template. For measurements in white matter region of interests (ROI), we applied a white matter template (JHU / ICBM WM atlas) and co-registered to the data set. Specific regions include bilateral uncinate fasciculus (UF, Figure 1) and sagittal stratum as a control. The registered ROIs were then transformed into each subject’s native space by applying the inverse of the spatial transformation estimated during normalization step, and mean FA, MD, AD, and RD values were computed.

Fractional anisotropy (FA) is a normalized measure that incorporates all three eigenvalues of diffusion tensor and is highly sensitive to the directional coherence of water diffusion. Mean Diffusivity (MD) is the average of the three diffusion tensor eigenvalues and is modulated by cellularity. Axial Diffusivity (AD) is the largest eigenvalue that is sensitive to the axonal structures. Lastly, Radial Diffusivity (RD) reflects diffusion perpendicular to the axons and may be modulated by myelination^59, 74^.

### Statistical Analysis

We first examined the relationship between twins’ anxiety and DTI measures after accounting for clustering within families using linear regression. Results from these individual-level analyses allowed us to determine if a relationship between UF and anxiety existed when genetic differences and other confounders were not controlled. Second, we adopted a twin difference design to examine the relationship between UF and anxiety. Capitalizing on the information provided by paired MZ twins increases the power to detect an association between two related variables (see Supplemental Material). Relative within-pair difference scores are calculated by randomly assigning one of the twins as Twin 1 and the other as Twin 2, and subsequently by subtracting the score of one twin from the score of the co-twin^75^. This method allows us to infer the directionality in detected associations between difference scores on anxiety and WM microstructure using the standard least squares regression framework:

$${\rm{\Delta }}Y={\beta }_{0}+{\beta }_{1}({\rm{\Delta }}X)$$

where ΔX and ΔY correspond to the anxiety difference and DTI difference score, respectively. Additionally, age and gender of the twin pairs were included as covariates in all models.

### Data availability

The datasets generated during and/or analyzed during the current study are available from the corresponding authors on reasonable request.

## Electronic supplementary material

Supplementary Information
