# Supplementary material for: Anxiety-related experience-dependent white matter structural differences in adolescence: A monozygotic twin difference approach
Source: Sci Rep. 2017 Aug 18;7:8749. doi: 10.1038/s41598-017-08107-6 (PMC5562810; doi:10.1038/s41598-017-08107-6)
Supplement: Supplementary file 1 — Supplementary Information [file 41598_2017_8107_MOESM1_ESM.pdf]

Supplementary material

Manuscript title

Anxiety-related experience-dependent white matter structural differences in adolescence: A monozygotic twin difference approach

Manuscript authors

Nagesh Adluru, Zhan Luo, Carol A. Van Hulle, Andrew J. Schoen
Richard J. Davidson, Andrew L. Alexander, H. Hill Goldsmith

This supplement provides additional details on

1. Theoretical demonstration of the power of pairing (pages 2–7).

2. Discussion on FA and AD vs. MD and RD (pages 8–12).

Theoretical demonstration of the power of pairing

Abstract
We show using mathematical arguments why discordance model can be statistically more powerful.

1 Introduction

We start with one of the simplest examples and build towards regression all using linear models. The basic flow would be

1. mean
   (a) single group
   (b) differences between two groups
      i. without pairing
      ii. with pairing
2. correlation
   (a) without paring
   (b) with pairing
3. genetic vs. environmental

2 Mean

2.1 Single group

Let's look at how mean for a measurement for a single group is estimated from a linear model point of view.

2.1.1 The model (abstract)

$$y = \beta_0 + \varepsilon, \quad \text{where } \varepsilon \in \mathcal{N}(0, \sigma). \tag{1}$$

2.1.2 The equations (data)

$$y_1 = \hat{\beta}_0 + \varepsilon_1, \tag{2}$$
$$y_2 = \hat{\beta}_0 + \varepsilon_2, \tag{3}$$
$$\vdots \tag{4}$$
$$y_n = \hat{\beta}_0 + \varepsilon_n. \tag{5}$$

2.1.3 The solution (empirical)

Since the system is overdetermined (more equations than unknowns) we can pick a "democratic" solution by just summing the left hand side and the right hand side of the equations as follows

$$y_1 + y_2 + \cdots + y_n = n\hat{\beta}_0 + \varepsilon_1 + \varepsilon_2 + \cdots + \varepsilon_n. \tag{6}$$
$$\Rightarrow \sum_{i=1}^{n} y_i = n\hat{\beta}_0 + 0 \quad ((\because E(\varepsilon) = 0)). \tag{7}$$
$$\therefore \hat{\beta}_0 = \frac{\sum_{i=1}^{n} y_i}{n}. \tag{8}$$

Notice that this is the classic "add-and-divide" formula.

2.2 Group differences

2.2.1 The model (abstract)

$$y = \beta_0 + \beta_1 \delta_{G_1}(x) + \beta_2 \delta_{G_2}(x) + \varepsilon, \quad \text{where } \varepsilon \in \mathcal{N}(0, \sigma), \tag{9}$$

$$\delta_K(x) = \begin{cases} 1, & \text{if } x = K \\ 0, & \text{otherwise} \end{cases}. \tag{10}$$

2.2.2 The equations (data)

Assuming you have n_1 samples from group G_1 and n_2 samples from G_2,

$$\boxed{\begin{aligned} y_1 &= \hat{\beta}_0 + \hat{\beta}_1 \cdot 1 + \hat{\beta}_2 \cdot 0 + \varepsilon_1, && (11)\\ y_2 &= \hat{\beta}_0 + \hat{\beta}_1 \cdot 1 + \hat{\beta}_2 \cdot 0 + \varepsilon_2, && (12)\\ &\vdots && (13)\\ y_{n_1} &= \hat{\beta}_0 + \hat{\beta}_1 \cdot 1 + \hat{\beta}_2 \cdot 0 + \varepsilon_{n_1}, && (14) \end{aligned}}$$

$$\boxed{\begin{aligned} y_{n_1+1} &= \hat{\beta}_0 + \hat{\beta}_1 \cdot 0 + \hat{\beta}_2 \cdot 1 + \varepsilon_{n_1+1}, && (15)\\ &\vdots && (16)\\ y_{n_1+n_2} &= \hat{\beta}_0 + \hat{\beta}_1 \cdot 0 + \hat{\beta}_2 \cdot 1 + \varepsilon_{n_1+n_2}. && (17) \end{aligned}}$$

2.2.3 The solution (empirical) without pairing

Again since the system is overdetermined (more equations than unknowns) we need a "democratic" solution again so we just use all pairs of points ($n_1 \times n_2$). Below we show a couple of pairs so we can see a pattern.

$$\begin{aligned} y_1 - y_{n_1+1} &= \hat{\beta}_1 - \hat{\beta}_2 + \varepsilon_1 - \varepsilon_{n_1+1}, && (18)\\ y_1 - y_{n_1+2} &= \hat{\beta}_1 - \hat{\beta}_2 + \varepsilon_1 - \varepsilon_{n_1+2}, && (19)\\ &\vdots && (20)\\ y_1 - y_{n_1+n_2} &= \hat{\beta}_1 - \hat{\beta}_2 + \varepsilon_1 - \varepsilon_{n_1+n_2}, && (21)\\ &\vdots && (22)\\ y_{n_1} - y_{n_1+1} &= \hat{\beta}_1 - \hat{\beta}_2 + \varepsilon_n - \varepsilon_{n_1+1}, \qquad \vdots && (23)\\ y_{n_1} - y_{n_1+n_2} &= \hat{\beta}_1 - \hat{\beta}_2 + \varepsilon_n - \varepsilon_{n_1+n_2}. && (24) \end{aligned}$$

Now by just summing left hand side (LHS) and right hand side (RHS) we get

$$n_2 (y_1 + y_2 + \cdots + y_{n_1}) - n_1 (y_{n_1+1} + y_{n_1+2} + \cdots + y_{n_1+n_2}) = n_1 n_2 \left(\hat{\beta}_1 - \hat{\beta}_2\right) + 0 \quad (\because E(\varepsilon) = 0). \tag{25}$$

$$\therefore \hat{\beta}_1 - \hat{\beta}_2 = \frac{n_2 \sum\limits_{i=1}^{n_1} y_i - n_1 \sum\limits_{i=n_1+1}^{n_1+n_2} y_i}{n_1 n_2}, \tag{26}$$

$$= \frac{\sum\limits_{i=1}^{n_1} y_i}{n_1} - \frac{\sum\limits_{i=n_1+1}^{n_1+n_2} y_i}{n_2}. \tag{27}$$

A key observation is that unless the ys from both groups are significantly separated the numerator is going to be small due to cancellations (see Fig. 1 (a)). Also notice again that this is just the classic "add-and-divide" within each group and taking a difference by simple subtraction.

2.2.4 The solution (empirical) with pairing

If we have the *a priori* knowledge that $n_1 = n_2$ and that y_1 corresponds to y_{n_1+1} etc. we can simply subtract the corresponding pairs and the solution would be[1]

$$y_1 - y_{n_1+1} = \hat{\beta}_1 - \hat{\beta}_2 + \varepsilon_1 - \varepsilon_{n_1+1}, \tag{28}$$

$$y_2 - y_{n_1+2} = \hat{\beta}_1 - \hat{\beta}_2 + \varepsilon_2 - \varepsilon_{n_1+2}, \tag{29}$$

$$\vdots \tag{30}$$

$$y_{n_1} - y_{n_1+n_1} = \hat{\beta}_1 - \hat{\beta}_2 + \varepsilon_2 - \varepsilon_{n_1+2}. \tag{31}$$

Again by just summing left and right hand sides we get

$$\hat{\beta}_1 - \hat{\beta}_2 = \frac{\sum_{i=1}^{n_1} (y_i - y_{n_1+i})}{n_1}. \tag{32}$$

Now we can observe that the requirement is that not *every* point in the other group has to be far from every other point. Just enough number of corresponding points have to be far (see Fig. 1 (b,c)).

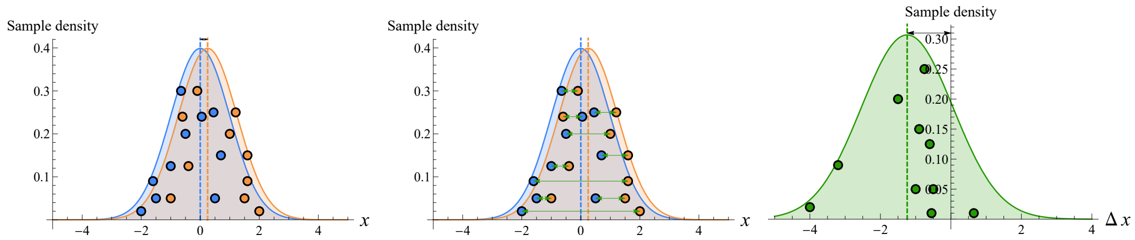

(a) (b) (c)

Figure 1: **No pairing vs. pairing. We can see that pairing helps enhance the effect size ("signal").** (a) Distribution of samples along a measure (x). There are two groups colored differently. A few random samples are shown. The effect size would be proportional to the distance between the two peaks.(b) By acknowledging the fact that there are dependencies between samples in the two groups we can look at the distribution of sample-pairs along the differential measure (Δx). (c) Distribution of sample-pairs along Δx. The effect size is proportional to the distance of the peak from 0.

3 Correlation

Now let's focus on identifying relationships between two measurements (x and y) (correlations) using linear models.

3.1 Correlation without pairing

3.1.1 The model (abstract)

$$y = \beta_0 + \beta_1 x + \varepsilon \quad \text{where } \varepsilon \in \mathcal{N}(0, \sigma). \tag{33}$$

[1] In a fully technical sense the model and the equations are also adjusted to reflect these correspondences and this simple subtraction is *forced* on us as the parsimonious approach. A full treatment of the correspondence effect is demonstrated in section 3 with the regression example.

3.1.2 The equations (data)

Let us say there are n samples collected in an experiment/study. Then

$$y_1 = \hat{\beta}_0 + \hat{\beta}_1 x_1 + \varepsilon_1, \quad (34)$$
$$y_2 = \hat{\beta}_0 + \hat{\beta}_1 x_2 + \varepsilon_2, \quad (35)$$
$$\vdots \quad (36)$$
$$y_n = \hat{\beta}_0 + \hat{\beta}_n x_1 + \varepsilon_n. \quad (37)$$
$$(38)$$

3.1.3 The solution (empirical)

We know from "high-school" that to a fit a line (in 2D space) we need two points. Here again we have many more points than necessary in other words the system of equations is overdetermined. Hence again we go with the "democratic" solution of using all pairs of points which in this case would be $n(n-1) = n^2 - n = O(n^2)$. Let us see a couple of pairs to see a pattern

$$y_1 - y_2 = \hat{\beta}_1(x_1 - x_2) + \varepsilon_1 - \varepsilon_2, \quad (39)$$
$$y_1 - y_3 = \hat{\beta}_1(x_1 - x_3) + \varepsilon_1 - \varepsilon_3, \quad (40)$$
$$\vdots \quad (41)$$
$$y_1 - y_n = \hat{\beta}_1(x_1 - x_n) + \varepsilon_1 - \varepsilon_n, \quad (42)$$
$$y_2 - y_3 = \hat{\beta}_1(x_2 - x_3) + \varepsilon_2 - \varepsilon_3, \quad (43)$$
$$\vdots \quad (44)$$
$$y_{n-1} - y_n = \hat{\beta}_1(x_{n-1} - x_n) + \varepsilon_{n-1} - \varepsilon_n. \quad (45)$$

Now simply summing the left and right hand sides again gives us

$$((n-1)y_1 + (n-2)y_2 + (n-3)y_3 + \cdots + y_{n-1}) - (y_2 + 2y_3 + \cdots + (n-1)y_n) \quad \text{LHS} \quad (46)$$
$$= \hat{\beta}_1 \left(((n-1)x_1 + (n-2)x_2 + (n-3)x_3 + \cdots + x_{n-1}) - (x_2 + 2x_3 + \cdots + (n-1)x_n)\right) \quad \text{RHS}. \quad (47)$$

$$\therefore \hat{\beta}_1 = \frac{\sum_{i=1}^{n-1}(n-i)y_i - \sum_{i=1}^{n-1} i y_{i+1}}{\sum_{i=1}^{n-1}(n-i)x_i - \sum_{i=1}^{n-1} i x_{i+1}} = \frac{\sum_{i=1}^{n-1}\sum_{j=i+1}^{n}(y_i - y_j)}{\sum_{i=1}^{n-1}\sum_{j=i+1}^{n}(x_i - x_j)}. \quad (48)$$

Again as in the case of mean if *every* pair of subtraction in the numerator and denominator are not consistently contributing there would be many cancellations and the value of $|\hat{\beta}_1|$ will be lower and many data points would not be in the confidence limits of the models. This effect is demonstrated in Fig. 2 (a). In the interest of brevity we only focus on $\hat{\beta}_1$ and not include $\hat{\beta}_0$ but similar observations and arguments can be made about that as well. In the next section we will see that by pairing we will relax that requirement to have consistency *only* among the predetermined pairs which is the fundamental reason for increase in the statistical power.

3.2 Correlation with pairing

3.2.1 The model (abstract)

$$y = \beta_0 + \beta_1 x + \beta_2 \text{Id} + \varepsilon, \quad \text{where } \varepsilon \in \mathcal{N}(0, \sigma). \quad (49)$$

3.2.2 The equations (data)

Let us say there are $2n$ samples (n pairs). Then

$$y_1 = \hat{\beta}_0 + \hat{\beta}_1 x_1 + \hat{\beta}_2 \text{Id}_1 + \varepsilon_1, \quad (50)$$
$$y_2 = \hat{\beta}_0 + \hat{\beta}_1 x_2 + \hat{\beta}_2 \text{Id}_2 + \varepsilon_2, \quad (51)$$
$$\vdots \quad (52)$$
$$y_n = \hat{\beta}_0 + \hat{\beta}_1 x_n + \hat{\beta}_2 \text{Id}_n + \varepsilon_n, \quad (53)$$

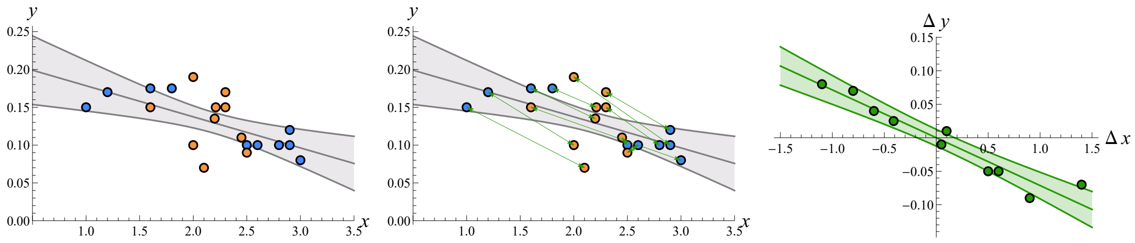

(a) (b) (c)

Figure 2: **No pairing vs. pairing. We can see that pairing helps enhance the effect size ("signal"). Similar to Fig. 1 but for regression.** (a) Correlation between two measures (x and y). The estimation does not take into account the pairwise dependency among the samples. The effect size would be proportional to the slope being away from 0 *and* the fit quality (samples within the 95% confidence band).(b) The pairwise dependencies are captured by the sloped arrows which capture rate of change of y (i.e. Δy) with respect x (i.e. Δx) for each pair of samples. (c) Linear model between Δx and Δy. We can clearly notice the enhancement in the effect size.

$$\boxed{\begin{aligned} y_{n+1} &= \hat{\beta}_0 + \hat{\beta}_1 x_{n+1} + \hat{\beta}_2 \mathrm{Id}_1 + \varepsilon_{n+1}, && (54)\\ y_{n+2} &= \hat{\beta}_0 + \hat{\beta}_1 x_{n+2} + \hat{\beta}_2 \mathrm{Id}_2 + \varepsilon_{n+2}, && (55)\\ &\vdots && (56)\\ y_{n+n} &= \hat{\beta}_0 + \hat{\beta}_1 x_{n+n} + \hat{\beta}_2 \mathrm{Id}_n + \varepsilon_{n+n}. && (57) \end{aligned}}$$

3.2.3 The solution (empirical)

Now we fit a "line" (technically a plane) in 3D space since there are three parameters that describe the linear model. Again the system is overdetermined and we will go with "democratic" solution. At first thought we would think we need to use all triplets of points to estimate the parameters. However we can take advantage of the fact that the third variable repeats to reduce the number of parameters to be estimated. By subtracting out the paired equations on left and right hand sides we get the following n equations

$$y_1 - y_{n+1} = \hat{\beta}_1(x_1 - x_{n+1}) + \varepsilon_1 - \varepsilon_{n+1}, \tag{58}$$

$$y_2 - y_{n+2} = \hat{\beta}_1(x_2 - x_{n+2}) + \varepsilon_2 - \varepsilon_{n+2}, \tag{59}$$

$$\vdots \tag{60}$$

$$y_n - y_{n+n} = \hat{\beta}_1(x_2 - x_{n+2}) + \varepsilon_1 - \varepsilon_{n+1}. \tag{61}$$

$$\tag{62}$$

Now simply summing the LHS and RHS gives us

$$\hat{\beta}_1 = \frac{\sum_{i=1}^{n}(y_i - y_{n+i})}{\sum_{i=1}^{n}(x_i - x_{n+i})}. \tag{63}$$

Here we simply require that the pairwise differences are consistent. This effect can be seen in Figs. 2 (b,c).

3.3 Genetic vs. environmental

Now that we have seen above how to go from an abstract model to an empirical solution and how using prior knowledge (in this case pairing) about the data in an experiment can help derive statistically more confident (less risky) empirical solutions let us see just in the abstract space how we can tease out the differential effects of environment (e) vs. genetic (g) on outcome variables in a twin design study. The basic model would be

$$y = \beta_0 + \beta_1 x + \beta_2 \mathrm{Id} + \varepsilon. \tag{64}$$

But let us say we know a priori that

$$x = \alpha_0 + \alpha_1 g + \alpha_2 e. \tag{65}$$

Combining Eqs. (64) and (65) we get

$$\begin{aligned} y &= \beta_0 + \beta_1(\alpha_0 + \alpha_1 g + \alpha_2 e) + \beta_2 \mathrm{Id} + \varepsilon, & (66)\\ &= \underline{(\beta_0 + \beta_1\alpha_0)} + \underline{\underline{\beta_1\alpha_1}} g + \underline{\underline{\underline{\beta_1\alpha_2}}} e + \beta_2 \mathrm{Id} + \varepsilon, & (67)\\ &= \underline{\psi_0} + \underline{\underline{\psi_1}} g + \underline{\underline{\underline{\psi_2}}} e + \beta_2 \mathrm{Id} + \varepsilon. & (68) \end{aligned}$$

Now just as we saw in section 3 we can get much better signal with

$$\Delta y = \psi_1 \Delta g + \psi_2 \Delta e, \tag{69}$$

where Δ here is the operator that subtracts *paired* measurements. But for monozygotic twins the assumption is that $\Delta g \approx 0$. Therefore we are really estimating the differential effects of environment on y. We just reaped two benefits (1) enhancing the statistical power by pairing (2) getting at the environmental effects.

3.3.1 Inference about the role(s) (α_2, ψ_2) of non-shared environmental differences (Δe)

First, we would like to note that the models described in here and used in our real experiments are *correlation* models and not causal/conditional models. The inference according to these models would be as follows. The non-shared environmental factors (Δe) are correlated with *both* Δx (measure of anxiety discordance, in our real experiments) as well as Δy (measure of uncinate fasciculus (UF) discordance, in our real experiments). We can see that algebraically as (assuming $\Delta g \approx 0$),

$$\begin{aligned} \Delta x &= \alpha_2 \Delta e, \quad \text{from Eq. (65) and} & (70)\\ \Delta y &= \psi_2 \Delta e, \quad \text{from Eq. (69).} & (71)\\ & & (72) \end{aligned}$$

But since we do not directly measure Δe in our studies, our equations in real experiments are derived from the model

$$\begin{aligned} \Delta y &= \underline{\psi_2}\left(\frac{\Delta x}{\alpha_2}\right), & (73)\\ &= \underline{\beta_1\alpha_2}\left(\frac{\Delta x}{\alpha_2}\right), & (74)\\ &= \beta_1 \Delta x. & (75) \end{aligned}$$

That is we can only estimate the role of non-shared environmental differences *indirectly* (via β_1) as the association between anxiety differences and UF differences, since we cannot estimate either α_2 or ψ_2. We would like to make a final note that these models represent phenomenological ansatz and based on further investigations and studies might have to adapted to include non-linear relationships between environmental factors and anxiety and white matter. □

Discussion on FA and AD vs. MD and RD

Abstract

We present material that elucidates differences and dependencies among the four commonly used DTI measures viz. fractional anisotropy (FA), axial diffusivity (AD), mean diffusivity (MD) and radial diffusivity (RD).

1 Introduction

Our starting point for this discussion is the diffusion tensor (D) which represents the diffusion (rate of area-of-spread[1]) of water molecules in the three standard orthogonal planes (with origin at the center of a voxel). It can be represented using a two dimensional matrix (also known as rank-2 tensor) as

$$D = \begin{bmatrix} D_{xx} & D_{xy} & D_{xz} \\ D_{yx} & D_{yy} & D_{yz} \\ D_{zx} & D_{zy} & D_{zz} \end{bmatrix}, \tag{1}$$

where D_{ij} refers to the diffusion in the plane spanned by orthogonal axes $\{i, j\}$. If $i = j$, then it refers to the square of the diffusion length along the axis i. Since the MR signal measured is not sensitive which side of the plane the water is diffusing, the matrix is symmetric i.e. $D_{ij} = D_{ji}$.

We will now,

1. dissect it and understand the commonly used DTI measures and
2. visualize to derive qualitative biological interpretations.

1.1 Dissecting the diffusion tensor

One could work directly with D if the goal was only getting at diffusivities in the standard planes. In fact if one were only interested in the average diffusivity known commonly as mean diffusivity (MD) or apparent diffusion coefficient (ADC), we can just read it off D as $\frac{D_{xx} + D_{yy} + D_{zz}}{3}$ due to a linear algebraic[2] fact that the trace of a matrix is equal to the sum of the eigenvalues of the matrix. Eigenvalues and eigenvectors capture one of the most basic symmetries[3] in vector spaces under linear transformations.

But one of the main goals of diffusion imaging is to reveal tissue microstructure including its orientation. Hence we need to extract which direction D is oriented in. A key linear algebraic tool that allows us to extract this is through eigen factorization (decomposition, dissection)[4] of D,

$$D = \begin{bmatrix} | & | & | \\ \mathbf{v_1} & \mathbf{v_2} & \mathbf{v_3} \\ | & | & | \end{bmatrix} \begin{bmatrix} \lambda_1 & 0 & 0 \\ 0 & \lambda_2 & 0 \\ 0 & 0 & \lambda_3 \end{bmatrix} \begin{bmatrix} | & | & | \\ \mathbf{v_1} & \mathbf{v_2} & \mathbf{v_3} \\ | & | & | \end{bmatrix}^{-1}. \tag{2}$$

[1] As a reference to the physical units, in a typical MRI scan, the observed diffusion in biological tissue without hindrance or restriction is on the order of 3 $\mu\text{m}^2{\cdot}\text{ms}^{-1}$.

[2] Linear algebra is the study of vector spaces under linear transformations. The notion of treating matrices as collections of vectors is a powerful convenience that allows us to glean patterns much more effectively compared to treating them just as a collection of numbers.

[3] Finding the set of $\mathbf{v}, \lambda$s that satisfy the equation $A\mathbf{v} = \lambda\mathbf{v}$ is essentially asking to find the vectors that remain the same (i.e. symmetric (invariant), upto scaling) under the transformation A.

[4] Another, now well known tool, called principal component analysis (PCA) which extracts the primary orientation of the spread of data fundamentally relies on the eigen decomposition of a covariance matrix.

Where $\mathbf{v_1}, \mathbf{v_2}, \mathbf{v_3}$ are the three main (orthonormal[5]) directions (in $\mathbb{R}^3$) of spread and the $\lambda_1, \lambda_2, \lambda_3$ are the extent of spread in those directions. Without loss of generality we can order the $\mathbf{v}$s and λs such that $\lambda_1 \geq \lambda_2 \geq \lambda_3$. The dissections of a sample diffusion tensor are shown in **Fig. 1**.

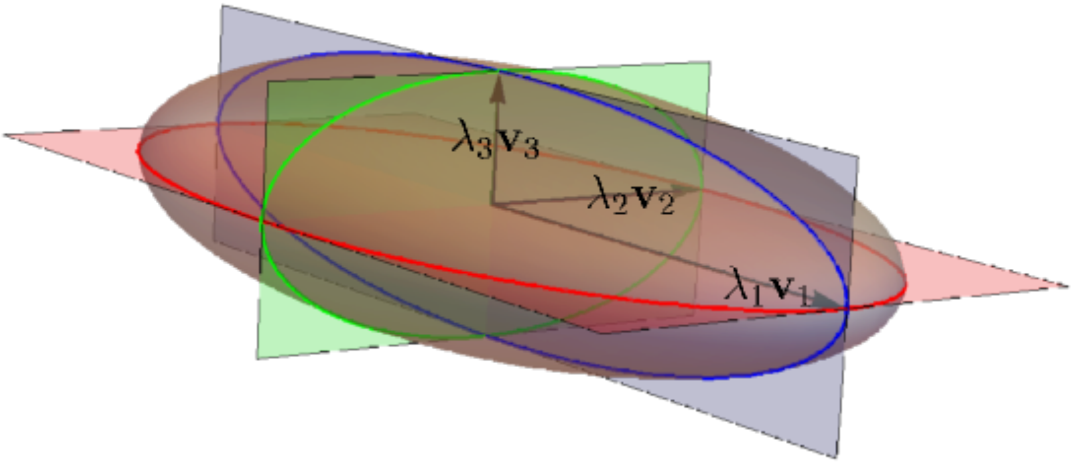

Figure 1: The dissected diffusion tensor. The three eigenvalues $(\lambda_1, \lambda_2, \lambda_3)$, eigenvectors $(\mathbf{v_1}, \mathbf{v_2}, \mathbf{v_3})$ and the three main planes of diffusion with the corresponding sections of the ellipsoids (in red, green and blue) are shown.

1.2 Understanding the dissected material (λs and $\mathbf{v}$s)

i) **Axial diffusivity (AD).** Let's first start with understanding the largest (**max**) of them all which is λ_1. This indicates the diffusivity in the direction of $\mathbf{v_1}$ which we can interpret as the primary orientation of the fiber in that voxel.

ii) **Radial diffusivity (RD).** If we just focus on diffusivity in the plane perpendicular to the main fiber orientation ($\mathbf{v_1}$), i.e. the plane spanned by $\mathbf{v_2}$ and $\mathbf{v_3}$), we have the two eigenvalues λ_2, λ_3 and their mean is $\frac{\lambda_2 + \lambda_3}{2}$ which is called the radial diffusivity (RD).

iii) **Mean diffusivity (MD).** Now let us focus on all the three eigenvalues (λs) jointly. If we have a collection of numbers and want to "understand" them we can start by computing their average (**mean**) (μ) which in this case is known as the mean diffusivity (MD)$= \frac{\lambda_1 + \lambda_2 + \lambda_3}{3}$.

iv) **Fractional anisotropy (FA).** Similarly we can also compute the (second order) variability in these measures (**variance**) (σ^2)

$$\sigma^2 = \frac{(\lambda_1 - \mu)^2 + (\lambda_2 - \mu)^2 + (\lambda_3 - \mu)^2}{3}. \tag{3}$$

Now if we want to normalize σ^2 such that its range is between 0 and 1, we would simply need to apply the following linear transformation,

$$\sigma^2_{\text{norm}} = \frac{\sigma^2 - \min(\sigma^2)}{\max(\sigma^2) - \min(\sigma^2)}. \tag{4}$$

$\sqrt{\sigma^2_{\text{norm}}}$ is the FA. $\min(\sigma^2) = 0$ since variance is always positive. Now let us calculate

[5] In general they are just required to be orthogonal but without loss of generality we can normalize them to be unit vectors.

max(σ^2) as this will reveal a few things about FA.

$$\sigma^2 = \frac{(\lambda_1^2 - 2\lambda_1\mu + \mu^2) + (\lambda_2^2 - 2\lambda_2\mu + \mu^2) + (\lambda_3^2 - 2\lambda_3\mu + \mu^2)}{3}, \tag{5}$$

$$= \frac{\lambda_1^2 + \lambda_2^2 + \lambda_3^2 - 2\mu \cdot (\lambda_1 + \lambda_2 + \lambda_3) + 3\mu^2}{3}, \tag{6}$$

$$= \frac{\lambda_1^2 + \lambda_2^2 + \lambda_3^2 - 2\mu \cdot 3\mu + 3\mu^2}{3}, \tag{7}$$

$$= \frac{\lambda_1^2 + \lambda_2^2 + \lambda_3^2 - 3\mu^2}{3}, \tag{8}$$

$$= \frac{\lambda_1^2 + \lambda_2^2 + \lambda_3^2}{3} - \left(\frac{\lambda_1 + \lambda_2 + \lambda_3}{3}\right)^2, \qquad \left[\because \mu = \frac{\lambda_1 + \lambda_2 + \lambda_3}{3}\right] \tag{9}$$

$$= \frac{3 \cdot (\lambda_1^2 + \lambda_2^2 + \lambda_3^2)}{3 \cdot 3} - \left(\frac{\lambda_1^2 + \lambda_2^2 + \lambda_3^2 + 2\lambda_1\lambda_2 + 2\lambda_1\lambda_3 + 2\lambda_2\lambda_3}{9}\right), \tag{10}$$

$$\left[\because (a + b + c)^2 = a^2 + b^2 + c^2 + 2ab + 2ac + 2bc\right] \tag{11}$$

$$= \frac{2(\lambda_1^2 + \lambda_2^2 + \lambda_3^2) - 2(\lambda_1\lambda_2 + \lambda_1\lambda_3 + \lambda_2\lambda_3)}{9}. \tag{12}$$

Now σ^2 is maximum when the numerator is maximum and that will happen when we do not subtract anything from $2(\lambda_1^2 + \lambda_2^2 + \lambda_3^2)$. That gives us,

$$\max(\sigma^2) = \frac{2(\lambda_1^2 + \lambda_2^2 + \lambda_3^2)}{9}. \tag{13}$$

$$\therefore \sigma^2_{\text{norm}} = \frac{\sigma^2}{\frac{2(\lambda_1^2 + \lambda_2^2 + \lambda_3^2)}{9}}, \tag{14}$$

$$= \frac{(\lambda_1 - \mu)^2 + (\lambda_2 - \mu)^2 + (\lambda_3 - \mu)^2}{3 \cdot \frac{2(\lambda_1^2 + \lambda_2^2 + \lambda_3^2)}{9}}, \qquad \text{[using Eq.(3)]} \tag{15}$$

$$= \frac{3(\lambda_1 - \mu)^2 + (\lambda_2 - \mu)^2 + (\lambda_3 - \mu)^2}{2(\lambda_1^2 + \lambda_2^2 + \lambda_3^2)}. \tag{16}$$

$$\therefore \text{FA}^{6} \equiv \sqrt{\sigma^2_{\text{norm}}} = \sqrt{\frac{3}{2}}\sqrt{\frac{(\lambda_1 - \mu)^2 + (\lambda_2 - \mu)^2 + (\lambda_3 - \mu)^2}{(\lambda_1^2 + \lambda_2^2 + \lambda_3^2)}}. \tag{17}$$

Now let us actually see when FA actually hits the maximum value. This will happen when

$$\lambda_1\lambda_2 + \lambda_1\lambda_3 + \lambda_2\lambda_3 = 0, \tag{18}$$

such that

$$\lambda_1^2 + \lambda_2^2 + \lambda_3^2 > 0. \tag{19}$$

Since we already have the order $\lambda_1 \geq \lambda_2 \geq \lambda_3 \geq 0$, this will happen only when $\lambda_1 > 0$ and $\lambda_2 = \lambda_3 = 0$ (i.e. fully restricted diffusion in the direction perpendicular to the fiber orientation). In fact it is precisely the ordering of the λs that imposes dependency (co-variability) between FA and AD. In some other words this dependency is by design, that is standard deviations (FA) of a set of numbers (eigenvalues) is driven higher by large values and by definition the large (eigen) values are called axial diffusivities. We also show this relationship using contour plots in **Fig. 2** so that we can easily see that FA gets higher when λ_1 (largest eigenvalue i.e. AD) is higher (and crosses) for fixed MD and RD.

1.3 Plausible biological interpretations

While pinning down biological interpretations of DTI measures definitively from *in vivo* data is quite difficult, we can provide some qualitative discussions of plausible changes to fibers when effects on FA and AD are detected but those on MD and RD are harder to detect. The biggest tool we have for this exercise is visualization of the DTI ellipsoids as a function of the eigenvalues

[6] This shows us clearly where the $\sqrt{\frac{3}{2}}$ factor is coming from, in the popular definition of the FA.

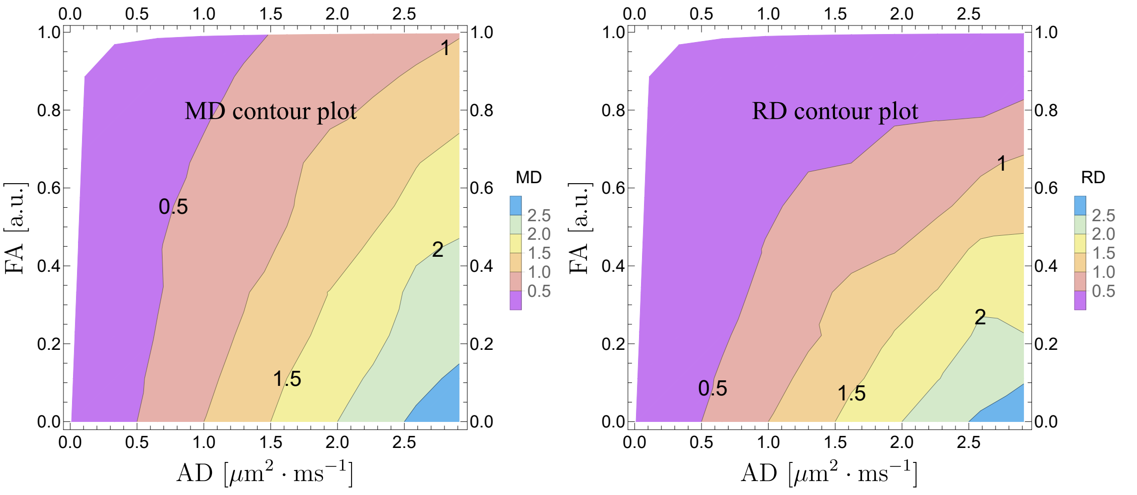

(a) MD as function of FA and AD.

(b) RD as function of FA and AD.

Figure 2: Contour plots (five contours are shown) of MD and RD as a function of FA and AD. We can notice from both the plots that for fixed values of MD and RD there is quite a bit of co-variability in FA and AD. More strongly so for contours of RD compared to those of MD, since λ_1 does not contribute to RD thus not saturating FA to its maximum as rapidly. More over nor these dependencies are linear nor monotonic (see for e.g. RD contour=2).

(λs). For this we need to pick a set of eigenvalues (λs) for which we have fixed MD and RD values, in other words we need to get the λs for MD and RD contours. **Fig. 3** shows these contours. Note that while **Fig. 2** shows the contours as a function of FA and AD, for visualization we need those as a function of the λs.

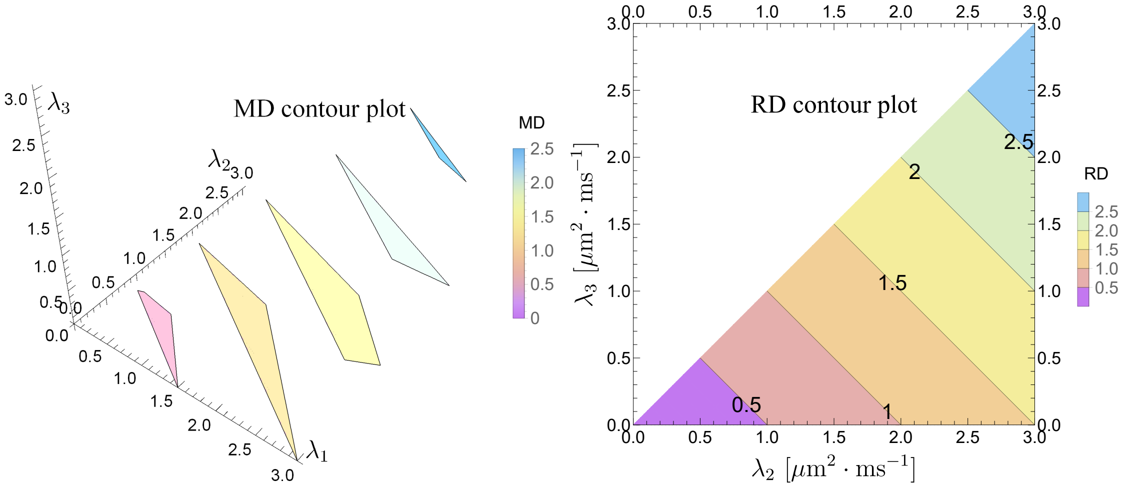

(a) MD as a function of $\lambda_1, \lambda_2, \lambda_3$.

(b) RD as function of λ_2, λ_3.

Figure 3: Contour plots of MD and RD as a function of λs. We enforce the $\lambda_1 \geq \lambda_2 \geq \lambda_3 \geq 0$. Five contours are shown. For MD we have contour surfaces, while for RD we have contour lines.

Fig. 4 (tob box) shows the shapes of the diffusion tensors for the MD contour at 1.5 and those for the RD contour at 0.5 are shown in **Fig. 4 (bottom box)**. These would be the examples of cases where one would be able to detect the effects on FA and AD but neither on MD nor on RD.

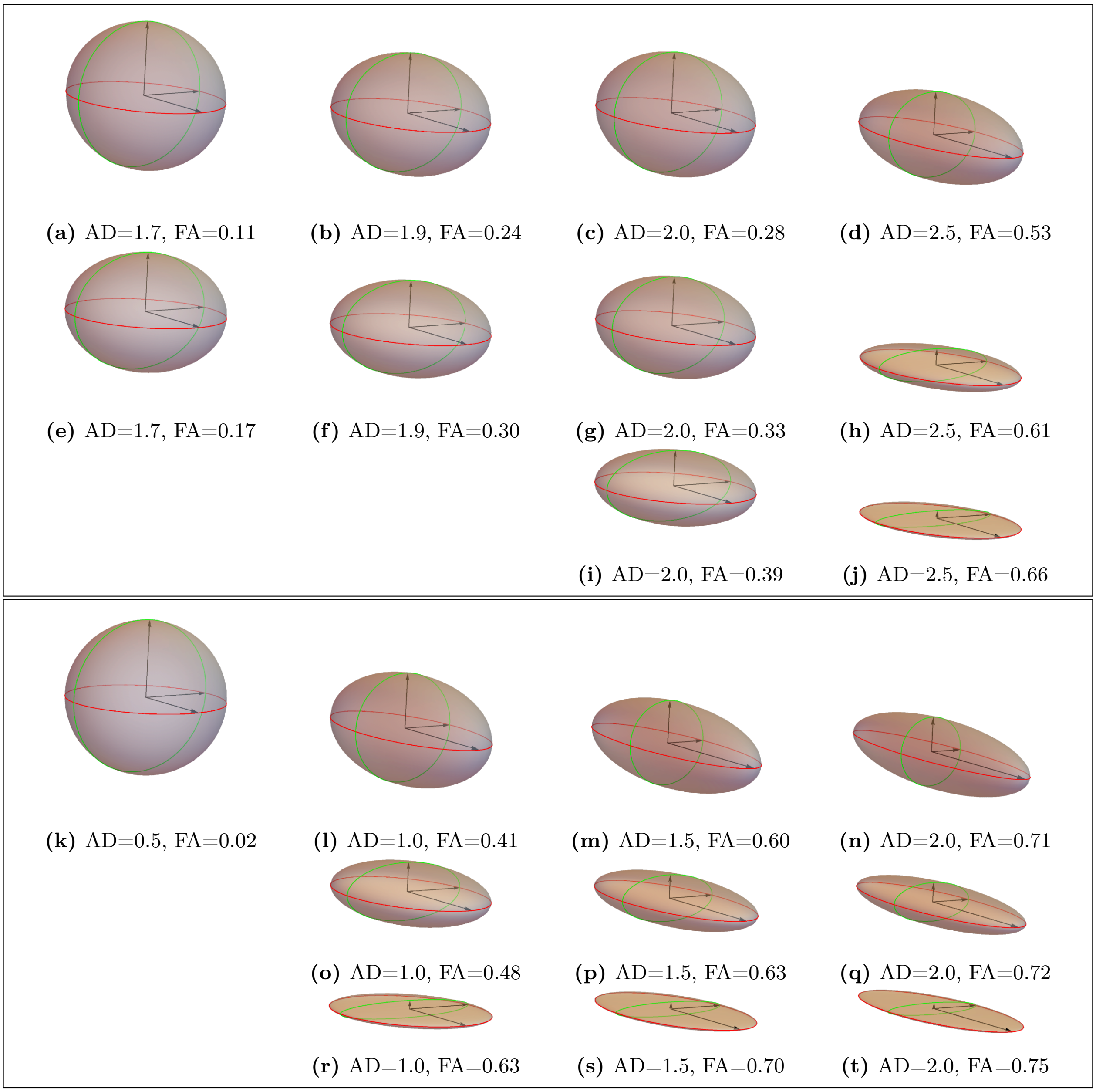

Figure 4: Sample shapes of the diffusion tensors along MD and RD contours. The corresponding AD and FA values are listed below the corresponding tensors. **Top box.** All the ellipsoids have same MD value of 1.5 $\mu m^2 \cdot ms^{-1}$. We can notice the "elongation" of tensors as AD increases and also increase in FA. The decrease in AD (and FA) as the anxiety measure increases could perhaps be related to some sort of "shrinkage" in the tract in a voxel. **Bottom box.** All the ellipsoids have same RD value of 0.5 $\mu m^2 \cdot ms^{-1}$. You can see that biologically the fiber can be in qualitatively quite different conditions. As AD and FA increases we can see a "squishing" effect along one of the directions perpendicular (here we are showing squishing in the direction of $\mathbf{v}_3$) to that of fiber orientation. The decrease in such an effect could perhaps be related to some sort of "unbinding" of the fibers in a voxel.
